# Impact of Fibrinogen Carbamylation on Fibrin Clot Formation and Stability

**DOI:** 10.1160/TH16-09-0704

**Published:** 2017-04-06

**Authors:** Veronika Binder, Brith Bergum, Stéphane Jaisson, Philippe Gillery, Carsten Scavenius, Endy Spriet, Anne Karin Nyhaug, Helen M. Roberts, Iain L. C. Chapple, Annelie Hellvard, Nicolas Delaleu, Piotr Mydel

**Affiliations:** 1 Broegelmann Research Laboratory, Department of Clinical Science, University of Bergen, Bergen, Norway; 2 University of Reims Champagne-Ardenne, Laboratory of Biochemistry and Molecular Biology, CNRS/URCA UMR N° 7369 MEDyC, Reims, France; 3 Interdisciplinary Nanoscience Center at the Department of Molecular Biology and Genetics, Aarhus University, Aarhus, Denmark; 4 Department of Biomedicine, University of Bergen, Bergen, Norway; 5 Periodontal Research Group MRC Centre for Immune Regulation, University of Birmingham, Birmingham, UK; 6 Malopolska Centre of Biotechnology, Jagiellonian University, Krakow, Poland; 7 Swiss Institute of Bioinformatics, Lausanne, Switzerland; 8 Faculty of Biochemistry, Biophysics and Biotechnology, Jagiellonian University, Krakow, Poland

**Keywords:** Fibrinogen, carbamylation, fibrin structure

## Abstract

Carbamylation is a non-enzymatic post-translational modification induced upon exposure of free amino groups to urea-derived cyanate leading to irreversible changes of protein charge, structure and function. Levels of carbamylated proteins increase significantly in chronic kidney disease and carbamylated albumin is considered as an important biomarker indicating mortality risk. High plasma concentrations and long half-life make fibrinogen a prime target for carbamylation. As aggregation and cross-linking of fibrin monomers rely on lysine residues, it is likely that carbamylation impacts fibrinogen processing. In this study we investigated carbamylation levels of fibrinogen from kidney disease patients as well as the impact of carbamylation on fibrinogen cleavage by thrombin, fibrin polymerisation and cross-linking *in vitro*. In conjunction, all these factors determine clot structure and stability and thus control biochemical and mechanical properties. LC-MS/MS analyses revealed significantly higher homocitrulline levels in patient fibrinogen than in fibrinogen isolated from control plasma. In our *in vitro* studies we found that although carbamylation does not affect thrombin cleavage per se, it alters fibrin polymerisation kinetics and impairs cross-linking and clot degradation. In addition, carbamylated fibrin clots had reduced fiber size and porosity associated with decreased mechanical stability. Using mass spectroscopy, we discovered that N-terminally carbamylated fibrinopeptide A was generated in this process and acted as a strong neutrophil chemoattractant potentially mediating recruitment of inflammatory cells to sites of fibrin(ogen) turnover. Taken together, carbamylation of fibrinogen seems to play a role in aberrant fibrin clot formation and might be involved in haemostatic disorders associated with chronic inflammatory diseases.

## Introduction

Post-translational modifications (PTMs) are (bio)chemical reactions in which amino-acid residues of proteins or peptides are covalently modified (1). That process significantly increases the structural and functional diversity of proteins introducing a complexity to the proteome that is several orders of magnitude greater than the coding capacity of the genome (2). PTMs are involved in many biological processes including enzyme activation, protein-protein interactions, protein transport and turnover. Many commonly observed PTMs are routinely tracked as disease markers while others may represent appropriate molecular targets for the development of specific therapies (2).

Carbamylation is a ubiquitous, non-enzymatic PTM, in which cyanate (OCN^–^) reacts with N-terminal amino groups (NH3^+^) of proteins or with lysine residues within polypeptide chains, to generate α-carbamyl amino acids and ε-carbamyl-lysine (homocitrulline) (3). Cyanate originates from the decomposition of urea and exists in plasma in equilibrium with its reactive form, isocyanic acid. In addition, the heme enzyme myeloperoxidase (MPO), released from activated neutrophils and monocytes, catalyses isocyanate production from thiocyanate at sites of inflammation (3, 4).

Under physiological conditions plasma urea concentration is too low to induce extensive carbamylation. However, various studies showed that conversion of lysine to homocitrulline in proteins occurs *in vivo* and that carbamylated proteins are involved in aging and different pathological conditions (5–8). In chronic kidney disease (CKD) urea concentrations can exceed 100 mM allowing for hypothetical cyanate concentrations of 1 mM (9). The fact that cyanate and isocyanic acid concentrations measured in CKD patients are in the range of 150 nM reflects the potential reactivity of cyanate particularly with plasma proteins as abundant as albumin and lipoproteins (10, 11). Since homocitrullination is irreversible over a proteins life span, long-lived extracellular matrix proteins are preferential targets for modification. This has been demonstrated *in vivo* by detection of carbamylated extracellular matrix proteins in the kidneys of renal patients using specific anti-homocitrulline antibodies (12). In accordance, accumulation of carbamylated proteins in various tissues was detected by LC-MS/MS in a mouse model of CKD (13). Structural changes induced by carbamylation commonly result in functional alterations of the proteins affected and has been described for collagen, matrix metalloproteinase-2, inhibitor of metalloproteinase-2, LL-37, the complement system and insulin (10, 14–17). Importantly, carbamylation is also recognised as a biomarker predicting the clinical outcome of kidney disease (10, 12, 18).

The discovery of anti-carbamylated protein antibodies (anti-CarP Ab) in rheumatoid arthritis (RA) and Sjögren’s syndrome provide further evidence of the physiological relevance of carbamylation *in vivo* (19). Several studies showed the presence of anti-CarP antibodies in anti-citrullinated protein antibody (ACPA) positive but also in ACPA negative RA patients. Anti-CarP Ab seropositivity was found predictive of a more severe clinical course and was therefore proposed as a novel serological marker for ACPA negative RA (5, 20). Importantly, anti-CarP antibodies present in RA patients are only partially cross-reactive with ACPA indicating that they constitute a distinct autoantibody system that recognises carbamylated but not citrullinated protein antigens (8, 21).

Fibrinogen is a 340 kDa dimeric glycoprotein composed of three pairs of polypeptide chains (Aα, Bβ and γ) that are stabilised by disulfide bonds (22). It is synthesised in hepatocytes and secreted into the blood with corresponding plasma concentrations of 1.5 to 3.0 g/l and a half-life of three days (23, 24). Spontaneous self-polymerisation of fibrinogen is prevented by the presence of short N-terminal sequences of the α- and the β-chains that are cleaved off by the serine protease thrombin in the course of the coagulation cascade exposing the polymerisation sites on the fibrin monomers. Concomitantly, thrombin activates the transglutaminase factor XIII that enables the conversion of the initially loose fibrin clot into the firm insoluble polymer capable of providing biophysical and biochemical support to the blood clot (25-27). Although activation of the coagulation pathway is essential for cessation of haemorrhage, tissue healing requires appropriate counterbalance of pro-coagulatory signals by the fibrinolytic system. The fibrin clot is essential for both, limitation of bleeding at sites of vessel injury and assembly and activation of the proteins involved in fibrinolysis. The key component of fibrinolysis is plasmin, another serine protease that catalyses the proteolytic degradation of fibrin resulting in formation of diffusible and soluble breakdown products including fragments D and E as well as D-dimers (25, 28).

The predominant function of fibrinogen is fibrin clot formation, though increasing evidence supports the involvement of fibrin(ogen) and its degradation products in the modulation of the inflammatory response. As a positive acute phase protein, increased plasma fibrinogen indicates a proinflammatory state and is associated with increased risk for the development of vascular inflammatory diseases including hypertension and atherosclerosis. Similarly, elevated concentrations of fibrin degradation products are used in clinical practice to detect coagulation hyperactivity and serve as predictors for thrombotic events (27). Fibrinopeptide B, released from fibrinogen upon fibrin formation can act as a chemoattractant for leukocytes and thereby directly control inflammatory reactions (26). In a recent publication, fibrinogen cleavage products were assigned a pivotal role in the pathophysiology of allergic asthma acting as inflammatory activators signalling via TLR-4. However, the findings describing specific inflammatory effects of fibrinopeptides are still inconclusive, indicating the need for more detailed investigations (23).

After albumin and immunoglobulins, fibrinogen is the third most abundant plasma protein. Considering that under inflammatory conditions, concentrations may increase three times above its physiological concentration, fibrinogen represents a primary target for PTMs that can affect enzymatic processing and cause the formation of dysfunctional haemostatic clots (30, 31). Within the fibrinogen sequence, lysine residues are located in close proximity to thrombin cleavage sites and polymerisation motifs and fibrin cross-linking is accomplished by formation of covalent bonds between glutamine and lysine residues within the α- and γ-chains (32). Moreover, lysine residues play a substantial role in plasminogen activation and plasmin-catalysed fibrin degradation (33).

The main objective of the present work was to determine the effects of fibrinogen carbamylation on the individual reaction steps leading to fibrin clot formation as well as on the immunomodulatory properties of the small peptide fragments generated in the course of the coagulation cascade. Establishing a mechanistic link between protein carbamylation and abnormal fibrin polymerisation as observed in a number of inflammatory diseases could lead to the discovery of disease-specific biomarkers. This would facilitate the identification of patients at risk for thrombosis or extended bleeding periods and potentially delineate an appropriate pathway for the development of new treatment strategies.

## Materials and methods

### *In vitro* carbamylation of fibrinogen

Plasminogen free fibrinogen (Merck Millipore, Darmstadt, Germany) was carbamylated by incubation with 1, 5 or 100 mM potassium cyanate (KOCN, Sigma Aldrich, Oslo, Norway) in PBS (pH 7.4) for up to 96 hours (h) at 37°C. The reaction was terminated by removal of excess KOCN using Amicon Ultra 2 ml centrifugal filter units (100 kDa cut-off, Merck Millipore) and fibrinogen concentrations were assessed on a Nano Drop ND-1000 spectrophotometer (Saveen Werner, Limhamn, Sweden) using an extinction coefficient (1 %, 1 cm, 280 nm) of 15.1 for fibrinogen. The purified samples were aliquoted and stored at -70°C until LC-MS/MS analysis or proteolytic cleavage by thrombin (Sigma Aldrich) was performed.

### Proteolytic cleavage of fibrinogen by thrombin

1 mg/ml unmodified and carbamylated fibrinogen (100 mM KOCN) were incubated overnight at 25°C with human thrombin (0.05 U/mg protein in the presence of 2.5 mM CaCl_2_) to induce fibrinopeptide release and fibrin polymerisation. Supernatants containing fibrinogen cleavage products were separated from the fibrin clots by two centrifugation steps (10 minutes [min] at 9600 × g followed by 5 min at 21,100 × g), immediately used in experiments or stored at -70°C for LC-MS/MS analysis.

### LC-MS/MS analysis of fibrinogen and fibrinopeptides

Unmodified and carbamylated fibrinogen (100 mM KOCN) were heat denaturated at 95°C for 10 min, reduced and alkylated in 20 mM Tris-HCl, pH 8 containing 5 mM DTT before iodoacetamide was added to a concentration of 15 mM. The reduced and alkylated samples were digested with trypsin (1:40 w/w) at 37°C for 16 h and the tryptic peptides were micropurified and lyophilised prior to mass spectrometric analysis. For MS-analysis, the peptides were dissolved in 5 % formic acid and loaded onto an EASY-nano LC system (Proxeon, Odense, Denmark) where they were separated using a ReproSil-Pur C18 AQ 3 µm reversed phase capillary column (Dr. Maisch, Ammerbuch-Entringen, Germany) packed in-house in a pulled emitter. The peptides were eluted using a gradient from 0 % to 35 % phase B (0.1 % formic acid, 90 % acetonitrile) over 30 min at 250 nl/min directly into a Triple TOF 5600+ mass spectrometer (AB Sciex, Les Ulis, France) equipped with a NanoSpray III source (AB Sciex).

### Identification of carbamylated lysine residues in fibrinogen

Collected MS files were converted to Mascot generic format (MGF) using AB SCIEX MS Data Converter beta 1.1 (AB Sciex) and the “proteinpilot MGF” parameters. The generated peak lists were searched against the swiss-prot database using in-house Mascot search engine (matrix science). Search parameters were chosen allowing one missed trypsin cleavage site and carbamidomethyl as a fixed modification. Carbamyl (K), pyro-Glu (N-terminal Q) and oxidation of methionine were selected as variable modifications. Peptide tolerance and MS/MS tolerance were set to 10 ppm and 0.1 DA, respectively.

### Determination of the relative amounts of released fibrinopeptides A and B by XIC

Relative amounts of fibrinopeptides were assessed based on the MS signal. The MS files were searched as described above and the Mascot.dat result files were used to generate a spectral library in Skyline (34). After data import, the chromatographic traces (extracted ion chromatograms) were manually inspected and adjusted to correct wrongfully assigned peaks. The relative abundance and standard deviation of proteins determined in all three of the technical replicates was calculated as the average MS intensity.

### Fibrin polymerisation kinetics

1.5 mg/ml unmodified and carbamylated fibrinogen (1 mM, 5 mM or 100 mM KOCN) in PBS containing 2.5 mM CaCl_2_ were pipetted into 96-well microtitre plates (Maxisorp Nunc-Immuno Plates, VWR, Oslo, Norway). Human thrombin (0.05 U/ml) was added immediately before start of the measurement and formation of the fibrin polymer was followed by recording the increase in OD (350 nm) for a period of 40 min on a SPECTRA max PLUS 384 plate reader (Molecular Devices, Sunnyvale, CA, USA). The specific thrombin inhibitor D-Phenylalanyl-prolyl-arginyl chloromethyl ketone (PPACK, Enzo Life Sciences, Oslo, Norway) was included to ascertain that the observed reaction was thrombin dependent.

Absorbance curves were characterised by the following parameters: 1) the slope at the steepest part of the polymerisation curve (Vmax), representing the rate of lateral protofibril association, 2) the time elapsed until an increase in absorbance of 0.01 units was detected (lag phase) and 3) the maximum absorbance (Max Abs) of the growing clot as a measure of the average thickness and density of the fibrin fibres.

### Factor XIIIa-catalysed fibrin cross-linking

50 µg unmodified and carbamylated fibrinogen (5 mM or 100 mM KOCN) in PBS containing 2.5 mM CaCl_2_ were incubated with human thrombin (0.05 U/mg protein) in the presence of factor XIIIa (0.5 U/mg protein, Nordic Diagnostica, Billdal, Sweden) for up to 2 h at 37°C. After addition of 5× Laemmli buffer (20 % DTT), samples were denaturated for 10 min at 100°C before aliquots corresponding to 10 µg protein were applied to Novex 8 % Tris Glycine protein gels (Life Technologies, Oslo, Norway). Separation was accomplished using Tris Glycine running buffer at an electric potential of 40 mA. Protein bands were visualised using Coomassie Stain for 1 h at 25°C followed by overnight destaining in a solution consisting of 140 ml acetic acid, 100 ml ethanol and 1760 ml dH_2_O.

### Fibrin polymer lysis

After incubation of 1 mg/ml unmodified and carbamylated fibrinogen (100 mM KOCN) with human thrombin (0.5 U/mg protein in the presence of 5 mM CaCl_2_) for 1 h at 25°C, plasmin (12.5 µg/mg protein, Sigma Aldrich) was added to the samples and the fibrin clots digested at 37°C for 0, 3, 6 and 9 h, respectively. Reactions were terminated by addition of 5× Laemmli buffer (20 % DTT) and plasmin inactivated at 70°C for 20 min. Aliquots of the digests corresponding to 10 µg protein were separated by SDS gel electrophoresis under the same experimental conditions as described above. The gel was stained using Biosafe Coomassie G-250 Stain and plasmin catalysed fibrin clot lysis was evaluated based on the intensity of the β-chain band.

### Scanning electron microscopy of fibrin polymers

Scanning electron microscopy (SEM) was used to investigate the surface structure of clots formed from unmodified and carbamylated fibrinogen (100 mM KOCN). Therefore 1 mg/ml fibrinogen was incubated overnight at room temperature with human thrombin (0.05 U/mg protein in the presence of 2.5 mM CaCl_2_) to induce fibrin clot formation. Clots were isolated by two subsequent centrifugation steps (10 min at 9600 × g followed by 5 min at 21,100 × g) and prepared for microscopic analysis. Therefore, samples were first fixed with 2.5 % glutaraldehyde in Sørensens buffer (pH 7.4) for 2 h and rinsed before they were treated with 1 % osmium (OsO4) for 1 h. After serial stepwise ethanol dehydration and critical point drying, samples were mounted on aluminium stubs and sputter coated with gold palladium. Fibrin clots were observed under a Field Emission SEM (Jeol JSM-7400 F, Jeol, Tokyo, Japan) and micrographs were recorded at an accelerating voltage of 4.0 kV.

### Chemotaxis assay

Chemotaxis of neutrophils in response to fibrinopeptides was studied using the Insall chamber (35). For each sample, discontinuous Percoll gradient-isolated neutrophils (400 µl in RPMI, final density 10^6^ neutrophils/ml) were added to acid washed (0.2 M HCl), dried and blocked (7.5 % BSA, 400 µl) coverslips (22 mm, VWR International, Radnor, PA, USA), which were incubated at 25°C for 30 min to allow the cells to adhere. The inverted coverslips were placed at the top of the chemotaxis chamber ensuring that the chemoattractant loading bays were exposed. 80 µl of the desired chemoattractant, fMLP (10 nM), IL-8/CXCL8 (200 ng/ml), fibrinopeptide A, fibrinopeptide B and carbamylated fibrinopeptide A (1 µM) or control (RPMI) was injected into the chemoattractant channels. The cell movement was analysed using a Zeiss Primovert microscope (Carl Zeiss Imaging, Thornwood, NY, USA) and images captured every 30 seconds for up to 40 frames per condition using a Q Imaging Retiga 2000R camera (Qimaging, Surry, BC, Canada).

### Fibrinogen Isolation from dialysis-patient and control plasma

Collection of blood samples from dialysis patients was approved by the Regional Committee for Medical and Health Research Ethics (REK vest), and informed consent was obtained in written from all participants. Control samples from healthy donors were collected at the blood bank (Department of Immunology and Transfusion Medicine, Haukeland University Hospital). Blood was taken at the start of dialysis and collected in citrate tubes. Plasma was isolated by centrifugation at 2100 × g and 25°C for 15 min and stored in 5 ml aliquots at -70°C. For fibrinogen isolation, plasma was thawed at room temperature overnight. Viral inactivation was carried out by adjusting the plasma to 0.15 % (v/v) tributyl phosphate and 0.5 % Triton X-100 followed by incubation at room temperature overnight. The samples were cooled to 4°C, adjusted to 10 % ethanol and incubated on ice overnight. After centrifugation at 3350 × g and 4°C for 20 min the resulting pellet was re-suspended in a re-suspension buffer containing 20 mM Tris, 55 mM sodium citrate and 27 mM lysine (pH 6.8). A second ethanol precipitation step was performed for 3 h on ice and the pellet re-suspended in a final volume of 2 ml re-suspension buffer. The solubilised protein was transferred into 150 mM NaCl using Amicon Ultra 2 ml centrifugal filter units (100 kDa cut-off), collected and centrifuged at 1500 × g and 25°C for 20 min. The protein concentration was determined according to the method of Bradford by spectrophotometric measurement at 595 nm including a BSA standard curve. Isolated fibrinogen was stored at -70°C until LC-MS/MS analysis.

### Evaluation of homocitrulline content in plasma-isolated fibrinogen samples

500 µl of samples containing 4 mg/ml of isolated fibrinogen were mixed with the same volume of 12 M HCl and subjected to acid hydrolysis in closed glass tubes for 18 h at 110°C after addition of 1 µM d7-citrulline and 65 µM d8-lysine (used as internal standards). Hydrolysates (1 ml) were twice evaporated to dryness under a nitrogen stream. Dried samples were resuspended in 100 µl of 125 mM ammonium formate and filtered using Uptidisc PTFE filters (4 mm, 0.45 µm, Interchim, Mannheim, Germany). Samples were then 10-fold diluted in 125 mM ammonium formate, a second dilution (1/20) being performed in 5 mM ammonium formate buffer (pH 2.9) for lysine quantification. Diluted hydrolysates were subjected to LC-MS/MS analysis (API4000, ABSciex) to quantify HCit and Lys. Liquid chromatography was performed using a Kinetex HILIC column (100 × 4.6 mm, 2.6 µm (Phenomenex, Le Pecq, France)) with 5 mM ammonium formate (pH 2.9) as mobile phase A and 100 % acetonitrile as mobile phase B. The flow rate was constant at 0.9 ml/min during all separation steps. Parameters for HPLC separation and mass spectrometry detection for homocitrulline and lysine assays were as previously described (13, 36).

### Statistics

As appropriate, statistical significances between group means were assessed using Mann-Whitney U test or One Way ANOVA and Tukey’s Post Test. All statistical analyses were performed using GraphPad Prism, version 6.0g and 7.0b for Mac (GraphPad, San Diego, CA, USA). P-values of < 0.05 were considered statistically significant.

## Results

### Carbamylation does not interfere with thrombin catalysed fibrinogen cleavage but leads to formation of structurally different cleavage products

Thrombin is activated by prothrombinase, a serine protease complex built up of factor Xa and its nonenzymatic cofactor Va assembled on the membrane surface of activated platelets. Catalytically active α-thrombin converts fibrinogen into fibrin by cleavage of the α- and the β-chains releasing fibrinopeptides A and B concurrent with exposure of the polymerisation motifs on both chains (GPR and GHRP). Upstream these signal sequences α- and β-chains contain lysine residues accessible for carbamylation.

**Figure 1:**
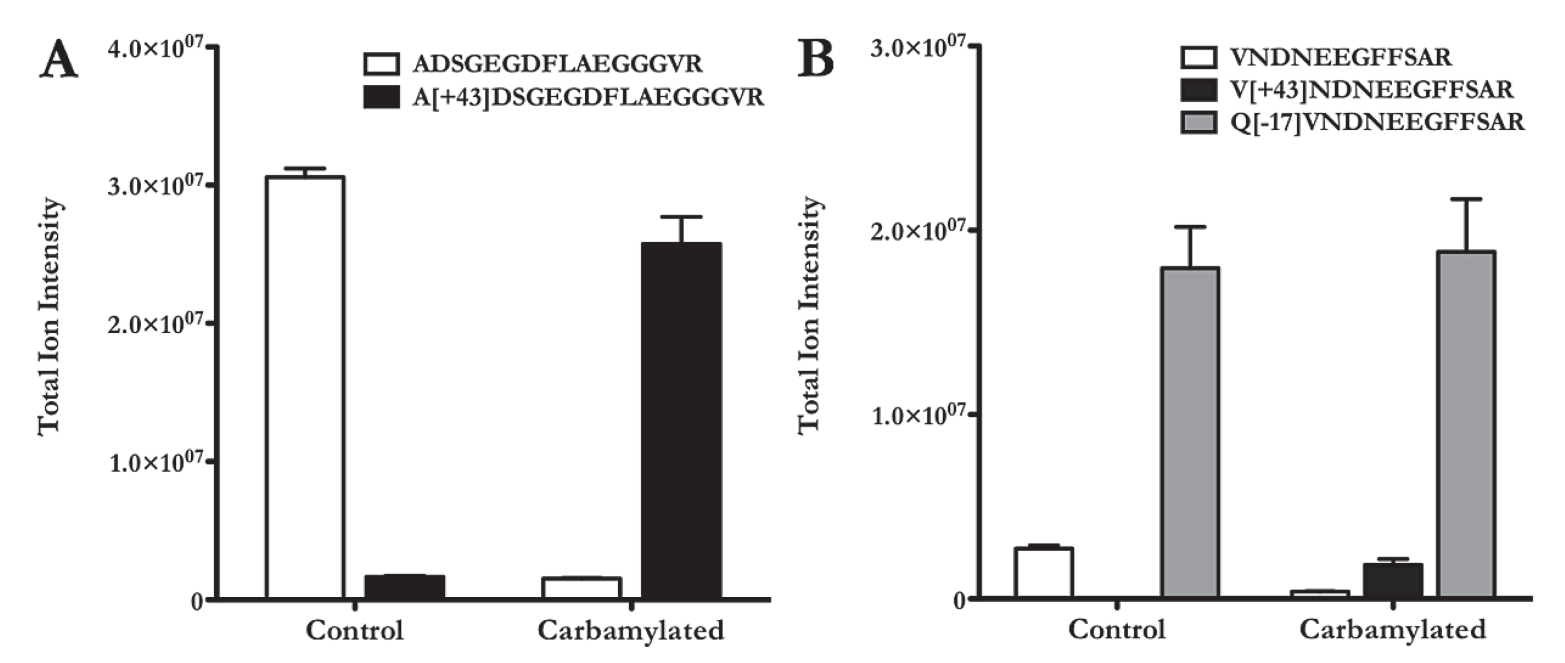
**Thrombin cleavage of carbamylated fibrinogen yields N-terminally carbamylated fibrinopeptide A.** Unmodified and carbamylated fibrinogen (100 mM KOCN) were acidified using trifluoroacetic acid (0.1 %), subjected to HPLC-MS analysis and the amounts of the different fibrinopeptides were assessed (relative estimation) based on the total ion intensities. Thrombin cleavage of unmodified and carbamylated fibrinogen leads to formation of comparable amounts of fibrinopeptide A, but in the modified samples the major part of it is N-terminally carbamylated (A). Pyroglutamination prohibits N-terminal carbamylation of fibrinopeptide B in carbamylated samples and unmodified controls (B). Bars represent mean + STD, n = 3.

By means of orbitrap/FTICR mass spectrometry we identified carbamylation on Lys29 of the α-chain and Lys22 of the β-chain of *in vitro* carbamylated fibrinogen. MS-analysis of the supernatants after fibrin clot formation containing the fibrinopeptides, and comparison of the total ion intensities revealed that carbamylation does not significantly alter the total amount of peptide-fragments generated. However, we found the N-terminal amino group of fibrinopeptide A to be highly carbamylated (►Figure 1 A). Interestingly, carbamylation of fibrinopeptide B was blocked by pyroglutamination of the N-terminal glutamine in carbamylated samples as well as in unmodified controls (Figure 1 B).

### Fibrinogen carbamylation alters fibrin clot formation

*In vivo*, formation of the polymeric fibrin network, together with platelet adhesion and aggregation is one of the key events in haemostasis (25). Carbamylation of positively charged side chain lysine residues in the individual fibrinogen chains leads to overall conformational changes with potentially profound effects on the reactions involved in fibrin polymerisation and thus on structure and properties of the resulting clot.

**Figure 2:**
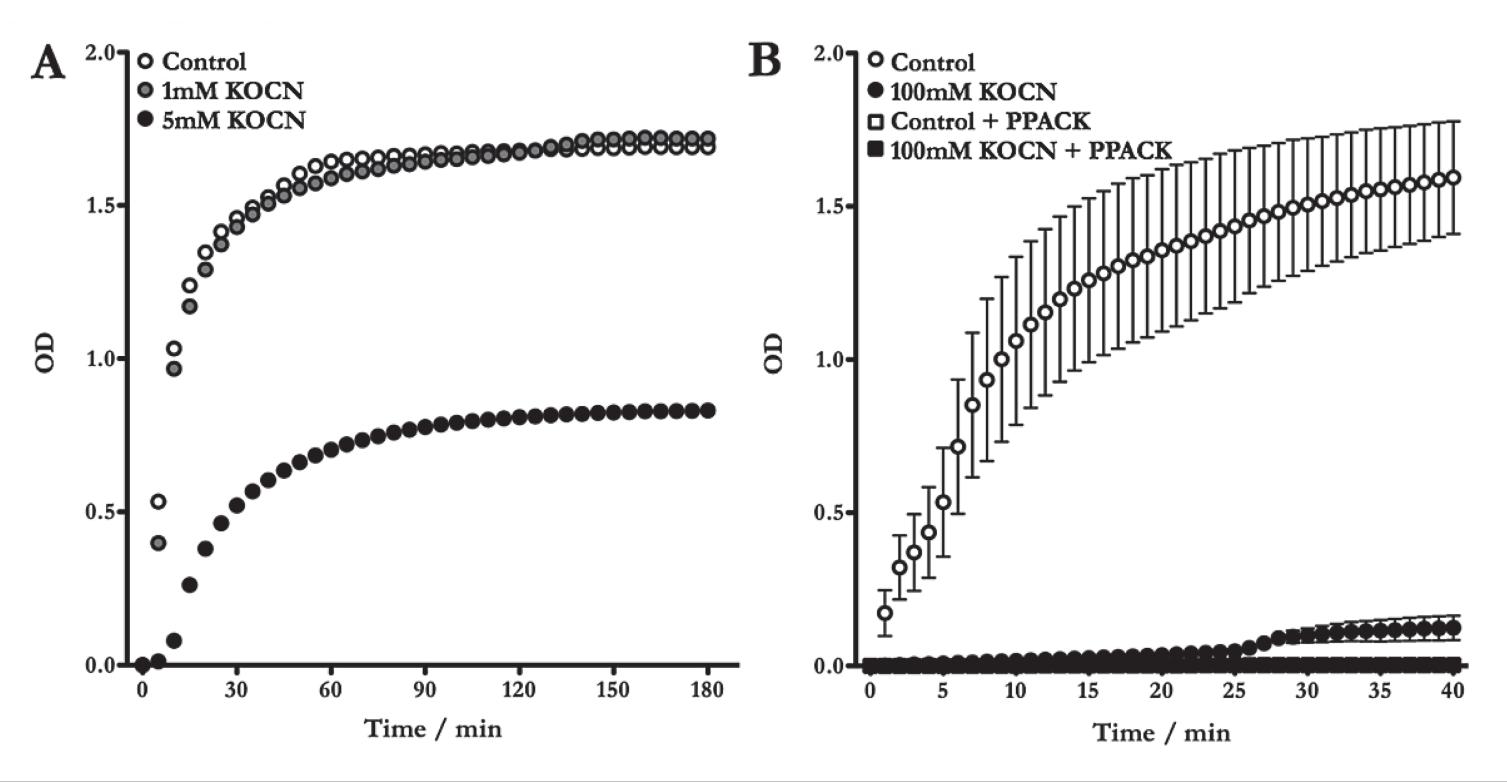
**Fibrinogen carbamylation alters fibrin polymerisation.** Unmodified and carbamylated fibrinogen ((A) 1 mM or 5 mM KOCN, (B) 100 mM KOCN) were treated with thrombin in microtitre plates and formation of the fibrin polymer was monitored by absorbance measurement on a plate reader (A). The specific thrombin inhibitor PPACK was included to confirm that the reaction was thrombin catalysed, which is evidenced by the reduction of the signal to base line in presence of the inhibitor (B). Curves show mean ± SEM, n = 2–3.

Representative curves from thrombin-catalysed fibrin polymerisation show that after carbamylation with 1 mM cyanate for 96 h, the course of reaction remains largely unchanged compared to the control. In contrast, carbamylation induced via addition of 5 mM cyanate for the same period of time significantly altered the kinetics of lateral protofibril aggregation (► Figure 2A). The change in polymerisation characteristics is evidenced by a prolonged lag time and a clear decrease in Vmax and Max Abs. Changes in these parameters reflect a delayed reaction start, a lower aggregation rate as well as different clot architecture as a consequence of carbamylation (► Table 1). Supporting the notion of a thrombin-dependent mechanism, addition of the specific thrombin inhibitor PPACK resulted in reduction of the signal to baseline level (► Figure 2B).

Comparable amounts of fibrinopeptides in carbamylated samples and controls (► Figure 1 A and B) together with the fact that in all samples clotting could be observed indicates that the decrease of the absorbance signal rather derives from different structural properties of the fibrin polymers than from a generally reduced clottablility of the protein. Fibrin clots from carbamylated fibrinogen appear transparent, loose and gel-like compared to the dense, rigid and opaque structures generated from the unmodified protein.

### Factor XIIIa catalysed cross-linking

Cross-linking refers to the introduction of covalent ε-(γ-glutamyl)-lysyl bonds between lysine residues and γ-glutamyl groups within the α- and γ-chains of fibrin by the transglutaminase factor XIIIa (32). According to Sobel et al. (37), the lysine residues listed in ►Table 2 are involved in cross-link formation.

Experimentally, fibrin cross-linking can be analysed by SDS-PAGE gel electrophoresis with subsequent visualisation of the reaction products specific for factor XIIIa catalysis.

**Figure 3:**
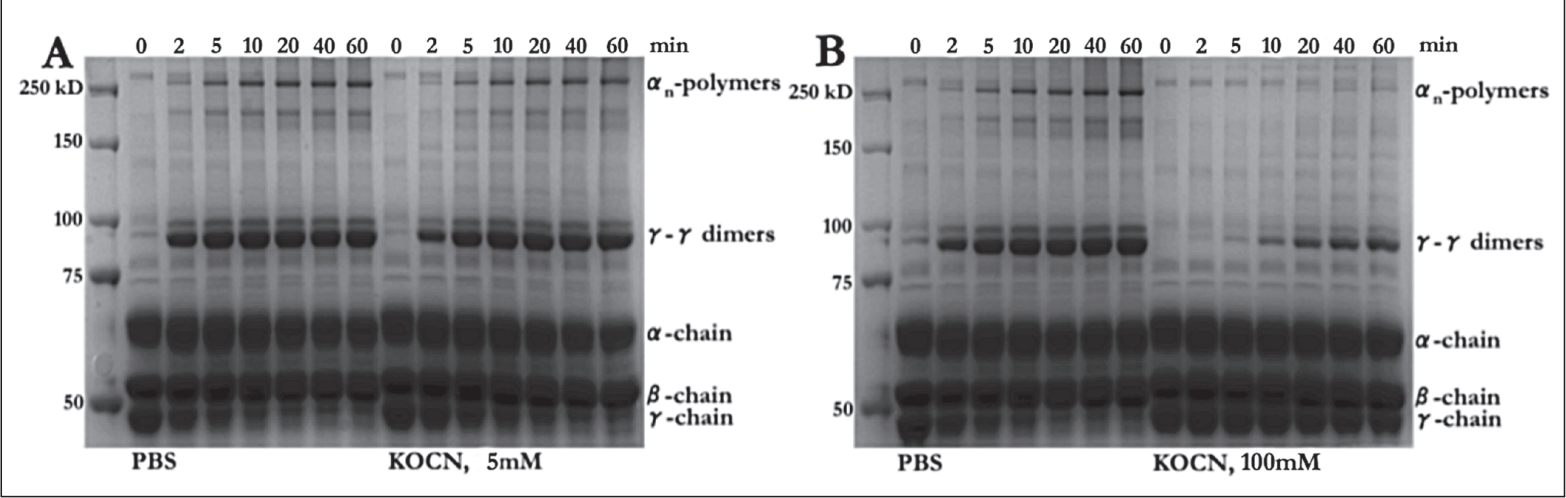
**Carbamylation of fibrinogen interferes with factor XIIIa mediated fibrin cross-linking.** Unmodified and carbamylated fibrinogen ((A) 5 mM, (B) 100 mM KOCN) were treated with thrombin and factor XIIIa for the indicated time periods before SDS gel electrophoresis was performed under reducing conditions and the gels were stained with Coomassie Stain. Formation of both, α-polymers and γ-dimers is impaired by fibrinogen carbamylation. While mild carbamylation conditions lead to delayed onset of the cross-linking reaction (A) clearly lower amounts of the reaction products are formed after same time periods in the case of more extensive modification (B). The findings are in accordance with the LC-MS/MS results suggesting that carbamylation would have a more profound effect on α-polymer than on γ-dimer formation.

Formation of α-polymers and γ-dimers was markedly delayed as a consequence of fibrinogen carbamylation using 5 mM cyanate for 96 h (►Figure 3 A). If fibrinogen was incubated in presence of 100 mM cyanate for 3 h prior to thrombin and transglutaminase treatment, α-polymers were practically undetectable and significant amounts of γ-dimers were only formed after 20 min (►Figure 3 B). These observations match the LC-MS/MS results showing that the α-chain lysine residues involved in cross-linking are in large part carbamylated contrary to the γ-chain residue, which we found to be unmodified in samples and controls (►Table 2). Altogether the findings indicate that carbamylation impairs fibrin cross-linking, which has detrimental consequences for the structural stability of the resulting fibrin clots.

### Fibrin clot structure

It has been documented that reaction steps involved in fibrin polymer formation determine the structure of the resulting clot and a decrease of the maximum absorbance was described in association with thinner and more compact fibres (38).

**Figure 4:**
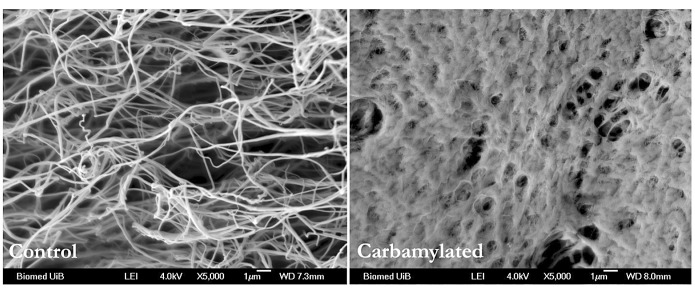
**Carbamylation alters the structure of the fibrin clot.** Samples of unmodified and carbamylated fibrinogen (100 mM KOCN) were incubated with thrombin, the resulting fibrin clots were prepared for microscopic analysis and images were recorded on a scanning electron microscope. Whereas clots from control fibrinogen have the characteristic fibrous structure, show clots prepared from the carbamylated protein an altered morphology resembling the thick matted layers found in association with a number of inflammatory conditions.

Scanning electron micrographs revealed that fibrinogen carbamylation has a clear impact on fibrin clot structure (►Figure 4). While clots generated from unmodified fibrinogen have the usual fibrous appearance, the clot structure observed in carbamylated samples resembles the thick matted layers built up of short and thin fibres that have been described in association with inflammatory conditions including RA, asthma, smoking and stroke (39). Moreover, microscopic images similar to ours were recorded from fibrin networks that were characterised as transparent, compressible and fragile (40). These are the same features that we found associated with fibrin polymers formed after thrombin treatment of carbamylated fibrinogen. Thus, carbamylation of fibrinogen appears to induce structural alterations that change mechanical and biochemical properties of fibrin clots.

### Carbamylation interferes with plasmin-catalysed fibrinolysis

Fibrinolysis is the process in which a fibrin clot is degraded in order to prevent uncontrolled growth and vessel blockade. Thereby, the main enzyme is plasmin, which cuts the fibrin network at specific locations generating circulating fragments that can be cleared by other proteases. Plasmin is synthesised as inactive plasminogen in the liver and is converted into its active form by either tissue-type plasminogen activator (tPA) or urokinase (uPA). Of the two enzymes, tPA seems to be of greater importance in terms of intravascular fibrin degradation (41). Plasminogen and tPA bind to the surface of the fibrin clot where plasminogen is activated to initiate fibrinolysis. Binding sites for tPA and plasminogen as well as plasmin cleavage sites involve lysine residues. As the fibrin network is degraded, C-terminal lysine residues become exposed serving as additional plasmin binding sites, thereby enhancing fibrinolysis (42).

Analysing the plasmin digests of fibrin clots generated from carbamylated and unmodified fibrinogen by SDS-PAGE, we found that the clots were clearly more resistant to plasmin induced lysis after carbamylation. This is evidenced by a marginal decrease in β-chain intensity in the carbamylated sample compared to the control where the respective decrease is obvious. Since the active form of plasmin was used in these experiments, the observed changes can only be related to the actual rate of plasmin catalysis and cannot be attributed to decreased binding of tPA or plasminogen. These results are in line with the more dense fibre arrangement and smaller pore size detected using SEM. Spatially, this may limit the movement of fibrinolytic enzymes through the fibrin network and therewith increase its resistance to fibrinolysis.

### Carbamylation converts fibrinopeptide A into a chemoattractant

Clot formation starts with activation of the intrinsic or extrinsic pathway of coagulation leading to prothrombin conversion to thrombin, which in turn catalyses fibrinogen processing and stimulates platelet activation. Once formed, the clot undergoes remodeling by proteolysis through the action of plasmin and neutrophil (PMN) elastase. The proteolytic fragments released in this process may in many ways control inflammation by mediating changes in vascular permeability, retraction and disorganisation of endothelial cells and inflammatory chemotaxis (43).

Human fibrinopeptide B, released from the fibrinogen β-chain by thrombin or contained within the primary plasmin cleavage product of fibrinogen Bβ 1–42, possesses chemotactic activity for neutrophils and fibroblasts. This finding implies that peptide products from fibrin(ogen) may have intrinsic biologic properties that extend beyond haemostasis.

To investigate if carbamylation can confer chemotactic activity to human fibrinopeptide A, which in its native form does not induce cell migration, we used an Insall chamber assay. This system enables observation of cell migration in real time. In the Insall chamber, cells migrate against a gradient of a chemotaxin thereby providing detailed information about their movement such as average speed of the cell over time in any direction, velocity (average speed of the cell over time in the direction of the chemotaxin) and the directional accuracy of chemotaxis (expressed as the chemotactic index).

**Figure 6:**
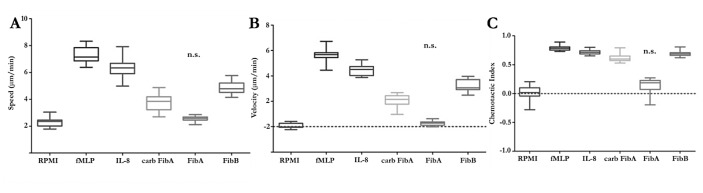
**Carbamylation converts fibrinopeptide A into a neutrophil chemoattractant.** Chemotaxis was analysed at a fibrinopeptide concentration of 1 µM. Neutrophil migration in response to the different fibrinopeptides was observed in the Insall chamber and values for speed, velocity and chemotactic index were evaluated. Carbamylation of fibrinopeptide A increased the migration speed of neutrophils in all directions (A), and directed towards the chemoattractant (velocity, B) as well as the directional accuracy of neutrophil chemotaxis (chemotactic index, C) compared to the unmodified peptide. The midline of each box represents the median value. Results were tested for statistical significance by One Way ANOVA and Tukey’s Post Test.

At a concentration of 1µM the neutrophil response to fibrinopeptide A was not different from the response to RPMI defined as the negative control. In contrast carbamylated fibrinopeptide A induced neutrophil chemotaxis as demonstrated by increased migration speed (►Figure 6 A), velocity (►Figure 6 B) and a higher chemotactic index (►Figure 6 C). Carbamylated fibrinopeptide A, however, was a weaker chemoattractant than native fibrinopeptide B. Nevertheless, these observations indicate that carbamylation alters the chemoattractant capacity of fibrinopeptide A and may therewith contribute to leukocyte recruitment to the sites of fibrin(ogen) turnover.

### Fibrinogen is carbamylated in the plasma of dialysis patients

**Figure 7:**
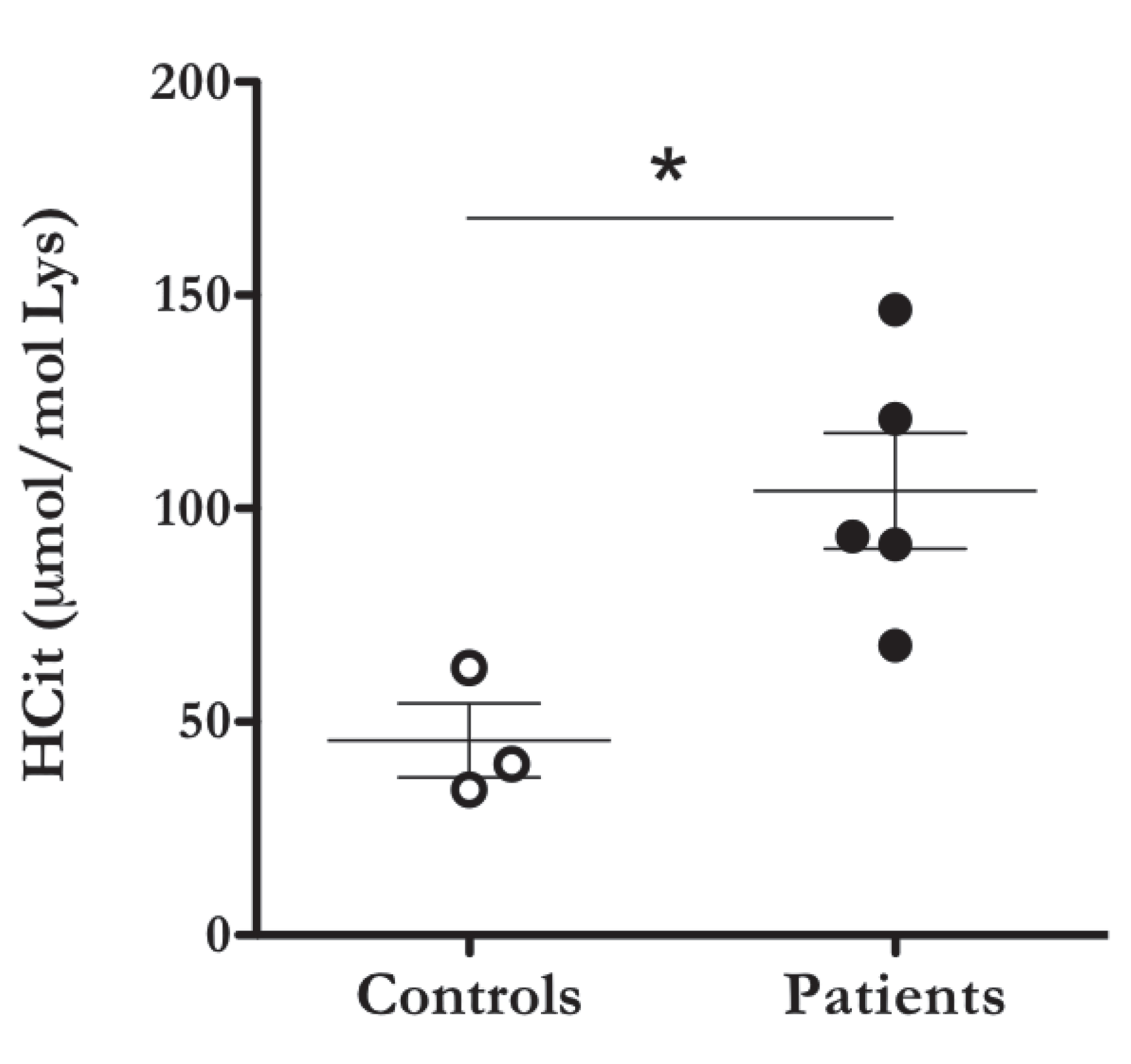
**Carbamylated fibrinogen is present in the plasma of dialysis patients.** Fibrinogen was isolated from plasma from dialysis patients and control subjects by ethanol precipitation and analysed by LC-MS/MS for homocitrulline quantification. Results are expressed as µmol homocitrulline per mol lysine. The statistical difference was evaluated by the Mann-Whitney U test. *P < 0.05 compared to controls.

Under the conditions of renal failure blood urea steadily accumulates generating a condition that requires treatment by intermittent haemodialysis or kidney transplantation. However, haemodialysis can only restore around 10 % of normal renal function leaving patients even when undergoing dialysis, with a chronic urea overload (uraemia) (8). Carbamylation of long-lived plasma proteins and protein components of the extracellular matrix is an unpreventable consequence. Various studies have shown that the irreversible addition of cyanate to proteins in the human body modifies cellular responses and thus underlies the development of CKD-associated complications such as accelerated atherosclerosis and inflammation (44). While it is well established that the level of carbamylated albumin is significantly higher in CKD patients than in controls and may thus serve as a quantitative biomarker for time-averaged urea concentrations, not much is known about the consequences of excessive carbamylation of other plasma proteins (44). Here we provide for the first time experimental evidence that fibrinogen is one of the targets for carbamylation in CDK patients. LC-MS/MS analysis of fibrinogen isolated from patient plasma showed significantly higher contents of homocitrulline than fibrinogen from control subjects (►Figure 7). Even though modification sites in patient and control fibrinogen were not determined, carbamylation clearly impacts charge and structure of the protein. Given that fibrin clot formation and fibrin(ogen) cell interactions require structural integrity, we speculate that fibrinogen carbamylation may be mechanistically involved in CKD-associated haemostatic dysfunctions.

## Discussion

The occurence of PTMs is one of the mechanisms to diversify the eukaryotic protein repertoire (2). Spontaneous non-enzymatic modifications of proteins, however, generate PTM-derived products that can act as endogenous toxins and are involved in a number of pathological conditions. Carbamylation results from binding of isocyanic acid to the a-NH2 extremity of proteins or the ε-NH2 groups of amino acid side chains thereby generating homocitrulline (18). *In vivo*, isocyanic acid is either formed by decomposition of urea or through oxidation of thiocyanate by the heme enzyme MPO released from activated neutrophils and monocytes.

Due to high urea concentrations and persistent low-grade inflammation, carbamylated proteins are found with increased prevalence in patients suffering from CKD and ESRD. Interestingly, the level of carbamylation in ESRD patients highly correlates with all-cause and cardiovascular mortality (44). One of the potential mechanisms responsible for the increased mortality may involve the detrimental effects of carbamylation on the protein components of lipoproteins. It had been shown that carbamylated LDL harbors atherogenic activities while carbamylation of HDL results in the loss of its anti-inflammatory and antioxidative properties (45).

Under physiological conditions, plasma concentrations of fibrinogen range from 1.5 to 3.0 g/l with a half-life of approximately three days. However, under the pathological conditions associated with vascular disruption, infection and inflammation, blood concentrations of fibrinogen can increase several fold (27). Thus elevated plasma concentrations, together with its comparatively long half-life, make fibrinogen a potential target for PTMs. Within the sequence of fibrinogen, lysine residues are located in close proximity to thrombin cleavage sites and polymerisation motifs. Moreover fibrin lysine residues are involved in cross-linking by factor XIIIa and are crucial for the binding of plasminogen to the fibrin surface as well as for plasminogen activation and plasmin-catalysed fibrinolysis (32, 46, 47). We and Scinocca et al. could show that most lysine residues of fibrinogen, including those in positions relevant for functional haemostasis, are accessible for carbamylation (48).

**What is known about this topic?**

- Various post-translational modifications of fibrinogen affect fibrin clot formation, structure and stability.
- Fibrinopeptide B, but not fibrinopeptide A, is chemotactic for neutrophils and monocytes.
- Plasma albumin is carbamylated in kidney disease patients and serves as a predictive marker of adverse clinical outcome.

**What does this paper add?**

- Specific effects of carbamylation on fibrin polymerisation kinetics, clot structure and resistance to fibrinolysis.
- Carbamylation converts fibrinopeptide A into a neutrophil chemoattractant.
- Fibrinogen represents a further plasma protein target for cyanate induced modification in kidney disease patients.

In this context, the aim of our study was to investigate the impact of carbamylation on the coagulation cascade, with respect to the reactions responsible for the conversion of fibrinogen into the insoluble fibrin clot.

We found that carbamylation did not affect fibrinogen cleavage by thrombin, as cleavage of unmodified and carbamylated fibrinogen yielded comparable amounts of fibrinopeptides A and B. A high degree of carbamylation was detected on the N-terminal amino group of fibrinopeptide A, while fibrinopeptide B was protected from modification by pyroglutamination of the N-terminal amino acid. Concomitantly with fibrinopeptide-release, thrombin catalysis leads to exposure of the polymerisation signals on the α- and β-chains (“knobs”), whose interaction with the complementary “holes” in the γ- and β-modules of a second fibrin monomer results in lateral protofibril aggregation. We followed the process of polymerisation of unmodified and carbamylated fibrinogen by turbidity measurement. In our experiments we saw a delayed onset of the polymerisation reaction (prolonged lag phase) and a reduced rate of fibre association after carbamylation of fibrinogen. Using LC-MS/MS we confirmed that the lysine residues located upstream the polymerisation signals on both chains were efficiently modified, suggesting that these structural alterations have an impact on fibrin monomer aggregation. However, since carbamylation has a far-reaching impact on protein structure, modification of more distant residues may also affect polymerisation kinetics. Proteolysis and polymerisation are not sequential but overlap temporally. It is conceivable that carbamylation decreases thrombin affinity to fibrinogen and decelerates fibrinopeptide release, which would also explain the different course of reaction. Our LC-MS/MS analyses were performed at a 1 h endpoint, when complete fibrin polymerisation is expected. Therefore we were not able to draw any conclusion on the early kinetics of the reactions. Interaction of the a-holes harboured within the γ-modules with the α-chain polymerisation sites is presumed to be the driving force of fibrin polymerisation. Carbamylation most likely induces conformational changes of the γ-chain C-terminus as well that have additional influence on polymerisation kinetics but the contribution of specific γ-chain modifications was not further assessed.

Polymerisation studies revealed that the structure of the fibrin network within the clot is heavily affected by carbamylation. To define structural differences in more detail, we analysed fibrin clots obtained after thrombin cleavage of unmodified and carbamylated fibrinogen using SEM. The images clearly show that carbamylation led to generation of fibrin aggregates consisting of very thin and subtle fibres forming a dense structure with smaller pores as compared to the characteristic fibrous polymer formed in the control sample. Morphologically, fibrin clots from carbamylated fibrinogen resemble the lace-like networks built up by short, thin and highly branched fibres observed in chronic inflammatory conditions such as RA, asthma, smoking and stroke. An increased rate of carbamylation is closely related to smoking and inflammatory diseases indicating that fibrinogen carbamylation represents one possible mechanism that contributes to the changes in fibrin clot architecture documented in association with these conditions.

Ten years ago, altered formation kinetics and structure of collagen fibrils after carbamylation have been reported. Similar to our studies of fibrinogen, carbamylation of collagen resulted in slower polymerisation associated with formation of a denser network composed of thinner fibres as compared to the unmodified protein (14). This indicates that the structural changes induced by carbamylation may be comparable for networking proteins. Nature and arrangement of fibrin fibres define biochemical and mechanical properties as well as the enzymatic stability of the clot. Hydrolysis of collagen by collagenase-1 (MMP-1) was impaired after carbamylation, showing that enzymatic degradation of proteins relates to their structural integrity (49). Dense clots that consist of tightly packed and highly branched small fibres as seen in carbamylated samples, have smaller pores that limit the movement of fibrinolytic enzymes leading to a reduction of the lysis rate. We observed that clots formed from carbamylated fibrin were digested significantly more slowly reflecting most probably decreased mobility of plasmin within the tight network and decreased capability of carbamylated fibrin to regulate the rate of fibrinolysis. Since active plasmin was used in these experiments, the impact of fibrin carbamylation on tPA and plasminogen binding as well as on plasminogen activation by tPA could not be evaluated.

The transglutaminase factor XIIIa introduces covalent bonds between the α- and the γ-chains of fibrinogen critically contributing to clot stabilisation. Using LC-MS/MS, we discovered that the majority of the α-chain lysine residues involved in cross-link formation were carbamylated. SDS-PAGE analysis revealed that carbamylation impairs cross-link formation. The absence of the stabilising covalent bonds could provide one explanation for the low mechanical stability of carbamylated fibrin.

Several studies have shown that peptides generated in the course of fibrin formation and fibrinolysis may modulate inflammatory responses via affecting leukocyte migration and cytokine production (23). Specifically, it has been reported that fibrinopeptide B delivers strong chemotactic signals to neutrophils and fibroblasts while no such effect was documented for fibrinopeptide A (43).

Considering the fact that we found structurally different fibrinopeptides after proteolytic cleavage of carbamylated and unmodified fibrinogen, we were interested to investigate if carbamylation could alter the chemotactic activity of fibrinopeptide A. To this end, we performed chemotaxis experiments with neutrophils in the Insall chamber using synthetic fibrinopeptides A (unmodified and N-terminally carbamylated) and B as chemoattractants. The graphs resulting from these experiments demonstrate that carbamylation converts fibrinopeptide A into a chemotaxin for neutrophils. Given that fibrinopeptides A and B are generated at the same sites, processing of carbamylated fibrinogen potentially leads to locally increased chemoattractant concentrations promoting leukocyte movement to the sites of fibrin(ogen) turnover.

Notably, we have demonstrated that in addition to already established targets as albumin and lipoproteins, fibrinogen is highly carbamylated in the plasma of dialysis-patients (44, 45). CKD-patients display a wide range of haemostatic dysfunctions that spans a pro-thrombotic tendency leading to excessive cardiovascular events as well as platelet dysfunctions leading to an increased bleeding tendency (50). The hypercoagulable state can be either linked to hyperfibrinogenaemia or qualitative changes of fibrinogen induced by oxidative stress, bilirubin or uraemic toxins. Modification of fibrinogen induced by oxidising and nitrating species including those produced by the neutrophil enzyme MPO could potentially contribute to pro-thrombotic properties of fibrinogen via different mechanisms (31, 51). Our *in vitro* experiments showed that fibrinogen carbamylation has profound effects on the structure of the fibrin clot and increases its resistance to fibrinolysis. It is probable, that *in vivo*, various modifications coexist within the same molecule and contribute to the overall functional changes of fibrinogen. Much evidence points to PTMs as pivotal contributors to increased thrombosis risk observed in CKD patients.

However, fibrinogen is also critically involved in primary haemostasis by linking adjacent platelets to a platelet aggregate (31). Carbamylation might affect the interactions of fibrinogen with its receptor on platelets, potentially leading to destabilisation of the platelet plug with prolongation of bleeding periods as a consequence.

The detection of specific antibodies against homocitrullinated fibrinogen in RA patients suggested the existence of carbamylated fibrinogen *in vivo*. This finding, however, may currently be qualified as indirect evidence as relevant targets of anti-CarP antibodies could not yet be clearly identified (5, 48). LC-MS/MS analysis of fibrinogen from RA patients may help to clarify if carbamylated fibrinogen in fact is a relevant antigen in RA *in vivo*.

In summary, our studies show that carbamylation impairs fibrin polymerisation and cross-linking and gives rise to altered clot morphology with reduced mechanical stability and increased resistance to fibrinolysis. Thus carbamylation of fibrinogen may be one underlying mechanism accounting for the formation of fibrin clots that are prone to the development of large pores under shear stress and to fragmentation. Decreased mechanical strength can lead to detachment of the clot from the vascular wall and induce embolism and ischaemia.

In contrast to the unmodified peptide, carbamylated fibrinopeptide A possesses chemoattractant properties for neutrophils potentially contributing to inflammatory cell recruitment to sites of fibrin deposition. As previously described, many proinflammatory functions of fibrin(ogen) and fibrin(ogen)-derived peptides are exerted through their interactions with immune cell receptors. The potential of carbamylation to modify receptor mediated signaling of fibrin(ogen) and fibrinopeptides in different cell types needs further investigation.

## Figures and Tables

**Figure 5:**
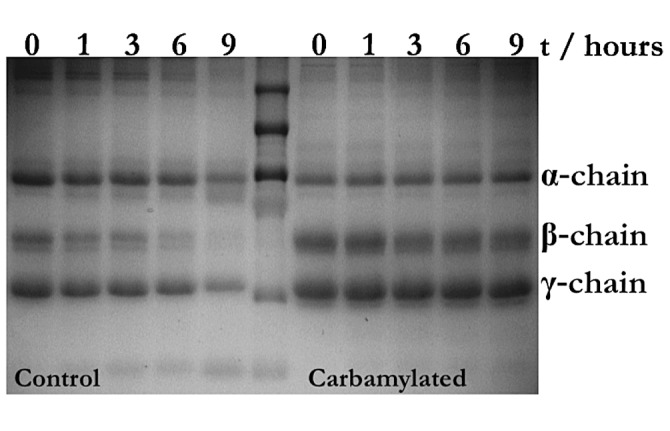
**Fibrin clots formed from carbamylated fibrinogen are more resistant to fibrinolysis.** Clots generated by treatment of unmodified and carbamylated fibrinogen (100 mM KOCN) with thrombin were digested with plasmin at 37 °C for the indicated time periods. After plasmin inactivation, aliquots of the digests corresponding to 10 µg protein were separated by SDS-PAGE under reducing conditions and the bands of the fibrinogen chains visualised by Coomassie Staining. Carbamylation increases the resistance of the fibrin clots to plasmin catalysed lysis as evidenced by an only moderate decrease in β-chain intensity over time compared to the control where the v-chain seems to be completely digested after 9 h.

**Table 1:** Parameters descriptive for the kinetics of fibrin polymerisation and the structure of the fibrin clots (based on the data graphically illustrated in Figure 2A).

	Lag Time (min)	Vmax (s^-1^) * 10^–2^	Max Abs
Control	< 1	10.33	1.691
1 mM KOCN	2–3	9.67	1.721
5 mM KOCN	4–5	2.99	0.831

**Table 2:** **Lysine residues listed in the table are reportedly involved in fibrin cross-link formation by factor XIIIa (37).** Residues in bold were found to be carbamylated by LC-MS/MS. Fibrinogen was carbamylated by treatment with 100 mM KOCN.

*a-chain*
Lys556, **Lys580**
**Lys418, Lys448**, Lys508, **Lys539**
**Lys208, Lys219, Lys224**, Lys427, **Lys429, Lys601**, Lys606
γ-*chain*
Lys406
